# Validation of a Tool to Evaluate Drug Prevention Programs Among Students

**DOI:** 10.3389/fpsyg.2021.678091

**Published:** 2021-06-18

**Authors:** Patrícia Paiva de O. Galvão, Juliana Y. Valente, Jacqueline N. Millon, Márcia H. S. Melo, Sheila C. Caetano, Hugo Cogo-Moreira, Jair J. Mari, Zila M. Sanchez

**Affiliations:** ^1^Department of Preventive Medicine, Universidade Federal de São Paulo, São Paulo, Brazil; ^2^Department of Clinic Psychology, Universidade de São Paulo, São Paulo, Brazil; ^3^Department of Psychiatry, Universidade Federal de São Paulo, São Paulo, Brazil; ^4^Department of Public Health, Hong Kong University, Hong Kong, China

**Keywords:** adolescents, primary prevention, surveys and questionnaires, validation studies, drugs

## Abstract

**Background:** School-based prevention programs have been implemented worldwide with the intention of reducing or delaying the onset of alcohol and drug use among adolescents. However, their effects need to be evaluated, being essential to use validated and reliable questionnaires for this purpose. This study aimed to verify the semantic validity and reliability of an instrument developed to evaluate the results of a government drug prevention program for schoolchildren called #Tamojunto2.0.

**Methods:** This is a mixed methods study with quantitative (test-retest, confirmatory factor analysis and non-response evaluation) and qualitative analyses (focus group and field cards). The self-administered questionnaires were used for a sample of 262 eighth-grade students (elementary school II) in 11 classes of four public schools in the city of São Paulo.

**Results:** The level of agreement was substantial (Kappa 0.60–0.79) or almost perfect (Kappa > 0.8) for almost all questions about the use of marijuana, alcohol, cigarettes, cocaine, crack, and binge drinking. The model fit indices, for almost all secondary outcomes, indicated that the modls underlying each scale, constituted by observed and latent variables, had a good fit adjustument. The focus groups and field cards provided high-quality information that helped the researchers identify the main difficulties in applying and understanding the questions.

**Conclusion:** The questionnaire showed high factorial validity, reliability and understanding by adolescents. After the necessary changes, identified in this study, the questionnaire will be suitable to evaluate the results of the #Tamojunto2.0 program in a randomized controlled trial.

## Background

School-based prevention programs have been implemented worldwide with the intention of reducing or delaying the onset of drug use among adolescents, with the most successful models being the ones working on the development of life skills and normative belief changes in relation to drug use (Strøm et al., 2014). To follow international prevention guidelines, the Brazilian Ministry of Health, in partnership with the United Nations Office on Drugs and Crime, decided to invest in the cross-cultural adaptation of a school program called Unplugged (Faggiano et al., 2010), which was renamed to #Tamojunto. Unplugged is a drug use prevention program for adolescents between 12 and 14 years of age, consisting of 12 classes that use interactive methods to work with social and personal skills, knowledge about drugs, and normative beliefs (Van Der Kreeft et al., 2009).

The randomized controlled trial (RCT), conducted in 2014 and 2015 to evaluate the effectiveness of #Tamojunto in the Brazilian context, showed an iatrogenic result for the onset of alcohol consumption, suggesting inadequate cultural adaptations in the components about alcohol (Sanchez et al., 2018). Considering these negative results, the material was readapted so that it would once again reflect the central elements of the European Unplugged (Faggiano et al., 2010). This new version, called #Tamojunto2.0, will be evaluated in the Brazilian population in 2019 and 2020 to substantiate the government's decision to expand it to all federation units.

Although the RCT that evaluated the effect of the first version of the program used data collection tools adapted and validated for the Brazilian population (Prado et al., 2016), after content adaptations and new international publications showing that drug programs should also be tested for mental health outcomes (Newton et al., 2018), it was decided to readapt the instrument. Considering the scarcity of validated instruments to evaluate prevention programs in schools (Strøm et al., 2014) and guarantee the validity of the study, it is necessary to have a consistent data collection methodology, which depends on adequate and precise measurement instruments (Neusa Maria Costa Alexandre Marina Zambon Orpinelli Coluci, 2011).

This study aimed to check the semantic validity and reliability of an instrument developed to evaluate the results of the #Tamojunto2.0 program to prevent drug use in students.

## Method

### Study Design

This is a mixed method study (Creswell, 2009) with quantitative and qualitative analyses to evaluate the semantic validity, construct validity and reliability of a self-administered questionnaire applied in the classroom for eighth-grade adolescents (Prado et al., 2016). The data were obtained through three procedures: (1) administering the questionnaire (74 questions) to students in the classroom, (2) focus groups with students after they filled the questionnaire, and (3) field notes regarding questionnaire administration in the classroom. The qualitative analyses emphasized the content of the questions presented by students and classroom procedures, while the quantitative analysis used (a) method of measurement stability (b) confirmatory factor analysis and (c) identification of the most difficult questions to answer through “non-response.” The stability of repeated responses was analyzed using two data collections with the same population and instrument, in a range of 15–20 days, through a “test-retest” evaluation. The collections occurred between September to November 2018.

This study was approved by the Ethics Committee of the Federal University of São Paulo, number 2.806.301, and registered in the REBEC (Clinical Trials Registry of the Ministry of Health)—-RBR-8cnkwq. The Consent to participate in the study was written and obtained from the schools' directors before randomization and from students, after randomization. All participants took part voluntarily after having given their free and informed consent based on the autonomy of adolescents guaranteed by the Brazilian Statute of the Child and Adolescent (Law No. 8069/1990). Moreover, parents were informed of the study by the directors and could recommend non-participation in data collection if they preferred. However, participation in the intervention was part of the school curriculum and was mandatory for all the students in the participating schools.

### Sample

The questionnaires were administered to a sample of 262 eighth-grade students distributed in 11 classes of four public schools in the city of São Paulo. The public schools were selected from different regions of the city to intentionally represent different socioeconomic, school performance, and activity length realities (part-time and full-time schools).

Sample size calculation considered the reproducibility analysis, taking as reference a 5% error and considering a type I error of 5%, a type II error of 10%, and a possibility of detectable disagreement of up to 20%. According to Temel and Erdogan (2017), the required sample would be 246 subjects for these defined characteristics.

### Procedures

The paper and self-administered questionnaire were distributed to students from all classes by trained researchers. On the first page of the questionnaire, the students created a secret code involving the generation of letters and numbers from personal information, which may only be decoded by the students themselves. These codes allow researchers to pair individual questionnaires at different study times and provide the participants anonymity and confidentiality, essential in a study on illicit behavior.

The administrator filled out a field form for each class, totaling 11 completed forms, which consisted of a semi-structured instrument on the school's general information, group behavior, conflict between students, class size, number of students present and absent in the classroom during administration, and refusals to participate. They also included an open field to describe any difficulties faced during the administration process and the students' doubts and questions.

Five focus groups were conducted after data collection using questionnaires in the classroom. Each focus group composed of 8–12 students, totaling 50 students in five groups, and two mediators (Stewart et al., 2007). The focus groups were audio-recorded and lasted a mean of 50 min. The classes were randomly chosen to participate in these focus groups with the objective of identifying difficulties in understanding the questions and the use of misinterpreted words or terms. The group mediator read the questions, and everyone discussed the understood meaning, unknown words, difficulties to answer, the layout, and the group's suggestions to improve the questionnaire.

The questionnaire data was entered into a custom typing system. The platform created could be accessed by several typists simultaneously and allowed real-time control of each typist's work and quality of typing. The internal coherence of the responses was assessed through consistency analyses to identify incomplete or incorrectly completed questionnaire on purpose.

### Instrument and Measurements

One of the basic instruments to formulate this questionnaire was tested by the EU-DAP (European Drug Addiction Prevention Trial) and used in previous studies on the effectiveness of Unplugged (Faggiano et al., 2010). The EU-Dap collects information, knowledge, and opinions on substance use, emphasizing alcohol, tobacco, and other drug use. In Brazil, this questionnaire was translated, adapted (Prado et al., 2016) and used to assess the first version of the #Tamojunto program (Sanchez et al., 2018) with some questions replaced by questions elaborated from two questionnaires widely used in several studies in Brazil: the questionnaire of the World Health Organization used by CEBRID (Brazilian Information Center on Psychotropic Drugs) in the VI Survey of drug use among students (Carlini et al., 2010), and the PENSE questionnaire (National School Health Survey) used by the Ministry of Health (IBGE, 2016).

Our instrument has 74 questions and, initially, there is a question about gender (male and female) and three questions about age, weight, and height data. The socioeconomic status was assessed by the scale of the Brazilian Association of Research Companies (Associação Brasileira de Empresas de Pesquisa—ABEP), a questionnaire with 15 dichotomous items (no/yes) (ABEP A. B. de empresas de pesquisa, 2018), which considers the head-of-household's education and the goods and services used, with scores ranging from 1 to 100 or in categories from A to E. Higher scores indicate a better economic status, and socioeconomic classes are ranked from A (highest) to E (lowest).

To the primary outcomes, there are 30 questions about the substance use prevalence, as detailed below:

1) Four items for alcohol use: lifetime use (no/yes), age of first drink (never drank or space to enter age), use in the past 12 months (no/yes), and use in the past 30 days with four response options (no, 1–5 days in the month, 6–19 days in the month and 20 days or more in the month).
2) Three items for binge drinking: lifetime episode (no/yes), episode in the past 12 months (no/yes) and episode in the past 30 days with 4 response options (no, 1 time, 2 times, and 3–5 times).
3) Four items for tabacco use: lifetime use (no/yes), use in the past 12 months (no/yes), use in the past 30 days with four response options (no, 1–5 days in the month, 6–19 days in the month and 20 days or more in the month) and how many cigarretes are smoked by day with 4 response options (I don't smoke, 1–10 cigarettes a day, 11–20 cigarettes a day or more than 20 cigarettes a day).
4) Three items for marijuana use: lifetime use (no/yes), use in the past 12 months (no/yes) and use in the past 30 days with four response options (no, 1–5 days in the month, 6–19 days in the month and 20 days or more in the month).
5) Three items for cocaine use: lifetime use (no/yes), use in the past 12 months (no/yes) and use in the past 30 days with four response options (no, 1–5 days in the month, 6–19 days in the month and 20 days or more in the month).
6) Three items for crack use: lifetime use (no/yes), use in the past 12 months (no/yes) and use in the past 30 days with four response options (no, 1–5 days in the month, 6–19 days in the month and 20 days or more in the month).
7) Three items for weight loss remedies use, without a medical prescription: lifetime use (no/yes), use in the past 12 months (no/yes) and use in the past 30 days with 4 response options (no, 1–5 days in the month, 6–19 days in the month and 20 days or more in the month).
8) Three items for tranquilizer remedies use, without a medical prescription: lifetime use (no/yes), use in the past 12 months (no/yes) and use in the past 30 days with 4 response options (no, 1–5 days in the month, 6–19 days in the month and 20 days or more in the month).
9) Three items about inhalants (inha some other product like glue, ether, paint remover, gasoline, benzine, enamel, acetone, thinner, turpentine, paint, aerosol deodorant for have a good sensation): lifetime use (no/yes), use in the past 12 months (no/yes) and use in the past 30 days with four response options (no, 1–5 days in the month, 6–19 days in the month and 20 days or more in the month).
10) One item about the use of other drugs not mentioned in the questionnaire (never used or space to enter the name of the drug).

In addition, the adaptation of this instrument to evaluate #Tamojunto 2.0 included other scales to measure seven secondary outcomes:

1) The Olweus Bully/Victim Questionnaire composed of two numerical scales, bullying suffered with eight dichotomous items (no/yes) and bullying practiced with nine dichotomous items (no/yes), to investigate school violence episodes (Solberg and Olweus, 2003). ***Bullying victimization***ranges from 0 to 8 (the higher the score, the more intense the bullying victimization) and ***bullying*** ***perpetration***ranges from 0 to 9 (the higher the score, the more intense the bullying perpetration). Each “yes” response is given 1 point, and a total score computed by summing. Scores above 1 are considered a positive screen for bullying.
2) The SCOFF (Sick/Control/One stone/Fat/Food) questionnaire is comprised of five dichotomous items (no/yes) to measure ***eating disorder symptoms***(Morgan et al., 1999). The SCOFF assesses five core aspects of anorexia (AN) and bulimia (BN). Each “yes” response is given 1 point, and a total score computed by summing. Scores above 2 are considered a positive screen for AN or BN.
3) The Stunkard Silhouette Scale consists of nine female figures and nine male figures, numbered 1–9, ranging from very thin to very obese (Scagliusi et al., 2006) to collect data from ***Body Satisfaction***. Participants were asked to choose one figure that they thought represented their body currently and one that they thought to be the ideal body. An ideal discrepancy score was calculated by diferença between answers.
4) The Strengths and Difficulties Questionnaire (SDQ) is comprised of 25 items with three answer options (false, more or less true and true) to collect data on ***psychiatric symptoms***(Goodman, 1997). The SDQ is a behavioral screening questionnaire and the 25 self-report SDQ items are divided between five scales of five items each: emotional symptoms, conduct problems, hyperactivity-inattention, peer relationship problems, and prosocil behaviors.
5) ***Excessive use of electronic games***was measured through nine dichotomous questions (no/yes), adapted from the DSM-5 (Diagnostic and Statistical Manual of Mental Disorders) (American Psychiatric Association, 2013).
6) ***Psychosis screening***was assessed using four questions with three answers options (not true, a little bit true and very true), adapted from the DSM-5 (American Psychiatric Association, 2013).
7) The data relating to ***Damage due to alcohol use was***adapted from EU-DAP questionnaire with 11 dichotomous items (no/yes) about problems attributed to the use of alcohol, such as fights, accidents, theft, among other problems.

The questionnaire also contains questions that evaluate the effect of program mediators, such as attitude, a scale with 10 dichotomous items (no/yes), decision making, a scale with nine dichotomous items (disagree/agree), beliefs about drugs, a scale with 11 items and four answer options (very likely, likely, unlikely and very unlikely), knowledge about drugs, a scale with six items and three answer options (no, yes and I do not know) and refusal skills, a scale with three items and four answer options (very likely, likely, unlikely, and very unlikely), all adapted from Eu-DAP questionnaire (Faggiano et al., 2010). However, it should be highlighted that the mediators were not analyzed regarding the reliability of the measurements in this study, but only regarding semantic understanding.

More information on the structure and source of the instrument is presented in Table 1.

**Table 1:** Details of questionnaire modules on knowledge, attitude, and behaviors related to alcohol, tobacco, and other drugs.

**Variable**	Main use^*^	**Source**
Use of drugs in life (first use, incidence, and prevalence) in the year and month (prevalence)	Primary outcome	CEBRID Carlini et al., 2010
Violence/bullying	Secondary outcome	Solberg and Olweus, 2003
Eating disorder symptoms	Secondary outcome	SCOFF Morgan et al., 1999
Body Satisfaction	Secondary outcome	Scale SilhouetteStunkart Scagliusi et al., 2006
Psychiatric symptoms	Secondary outcome	Goodman, 1997; Fidalgo et al., 2016; (CEBRID study)
Psychosis screening	Secondary outcome	DSM-5 American Psychiatric Association, 2013
Excessive use of electronic games	Secondary outcome	DSM-5 American Psychiatric Association, 2013
Damage due to alcohol use	Secondary outcome	EUDAP Faggiano et al., 2008
Socioeconomic class	Confounder	ABEP A. B. de empresas de pesquisa, 2018
Demographic data	Confounders	CEBRID Carlini et al., 2010
Attitude	Mediator	EUDAP Faggiano et al., 2008
Beliefs on drugs	Mediator	EUDAP Faggiano et al., 2008
Decision making	Mediator	EUDAP Faggiano et al., 2008
Knowledge on drugs	Mediator	EUDAP Faggiano et al., 2008
Refusal skill	Mediator	EUDAP Faggiano et al., 2008

* *A same variable may have more than one type of use in secondary analysis*.

*CEBRID, Brazilian Information Center on Psychotropic Drugs; SCOFF, Sick/Control/One stone/Fat/Food; DSM, Diagnostic and Statistical Manual of Mental Disorders; EUDAP, European Drug Addiction Prevention Trial; ABEP, Brazilian Association of Research Companies; PENSE, National School Health Survey; IBGE, Brazilian Institute of Geography and Research*.

### Data Analysis

#### Quantitative Analysis

The reliability of the instrument regarding primary outcomes (frequency of drug use) was analyzed using Kappa's statistics, which measures the consistency level of categorical responses at two moments of questionnaire administration, in addition to the agreement that would be randomly expected. Therefore, the questionnaire was applied to the same students, with a 2–3-week interval, with answers being matched using the Levenshtein algorithm, which identifies similarities between a character set for comparative purposes. The Kappa test was analyzed for consistency in both administration times, with almost perfect (> 0.79), substantial (0.60–0.79), moderate (0.40–0.59), regular (0.20–0.39), and low (0.0–0.19) (Landis and Koch, 2012).

Using the Mplus 7.4 statistical modeling program, we conducted confirmatory factor analysis (CFA), to provide evidence for the construct validity of secondary outcomes scales (bullying, eating disorder symptoms, psychiatric symptoms, psychosis screening, excessive use of electronic games and damage due to alcohol use). To assess the quality of fit indices, the comparative fit index (CFI), the Tucker-Lewis index (TLI), the weighted root mean square residual (WRMR), and the root mean square error (RMSEA) approximation were used. The cutoff criteria used to determine the quality of fit were CFI and TLI near or above 0.90, WRMR near to 1.0 and RMSEA near or below 0.08 (Little et al., 2013).

The reliability of secondary outcomes (bullying, eating disorder symptoms, psychological symptoms, and excessive use of electronic games) was assessed through the Cronbach's Alpha test at both the data collection moments, since these measurements used continuous outcomes collected through scales. This method is considered conservative, especially in cases where the items on the scale are heterogeneous, dichotomous, or define multifactorial structures. This test estimates the internal consistency of item variances and test totals of subject that are classified as excellent (> 0.9), good (> 0.8), acceptable (> 0.7), questionable (> 0.6), low (> 0.5), and unacceptable (<0.5) (Maroco and Garcia-Marques, 2006).

The proportion of answers left blank was evaluated in all questionnaires as a suggestive measure of the difficulty of the questions, that is, each question was subjected to a descriptive analysis to assess the proportion of adolescents not answering it.

#### Qualitative Analysis

Qualitative data analysis aimed at checking the semantic and operational validity of #Tamojunto2.0 questionnaire through the analysis of field cards and focus groups. The analysis strategy chosen was the data based theory, due to its richness to construct categories of analysis based on the studied context (Strauss and Corbin, 2008), which is appropriate to clarify difficulties reported by students in answering the questionnaires. After one researcher transcribed the groups' audios, another researcher read this transcript in depth and summarized each focus group, and another member of the research group analyzed these summaries highlighting common situations between groups. Thus, it was possible to determine three main categories of analysis: (1) semantic difficulties, (2) difficulties related to the structure and layout of the questions, and (3) environment and logistics characteristics that facilitated or hindered questionnaire answering.

## Results

### Study Population Profile

During the first collection, 262 students answered the questionnaire, with 257 students answering it during the second collection. A total of 207 questionnaires were paired on both occasions using the Levenshtein algorithm. Considering as base only the students present on the 1st day of data collection, the proportion of paired questionnaires was 79%. The participants had a mean age of 13.74 years (SE 0.05), with a predominance of females (53.3%) and socioeconomic class B and C (37.02 and 46.56%, respectively) (Table 2).

**Table 2:** Sociodemographic characteristics of public-school students in the city of São Paulo participating in the validation study of the #Tamojunto drug prevention program evaluation questionnaire (*N* = 262).

**Sociodemographic characteristics**	***n*/*m***	**%**	**SE**	**CI**
**Sex**				
Girl	138	53.28	3.11	[47.15; 59.32]
Boy	121	46.72	3.11	[40.68; 52.85]
Mean age	13.74		0.05	[13.63; 13.84]
**Age**				
12	1	0.38	0.38	[0.05; 02.69]
13	101	38.55	3.01	[32.81; 44.62]
14	140	53.44	3.09	[47.33; 59.43]
15	17	6.49	1.52	[4.06; 10.21]
16	2	0.76	0.54	[0.19; 03.03]
17	1	0.38	0.38	[0.05; 02.69]
ABEP^*^classification				
A	17	6.49	1.52	[4.06; 10.22]
B	97	37.02	2.99	[31.35; 43.07]
C	122	46.56	3.09	[40.56; 52.66]
D-E	26	9.92	1.85	[6.83; 14.21]
**Schools**				
1	65	24.81	—	—
2	31	11.83	—	—
3	60	22.90	—	—
4	106	40.46	—	—

* *Brazilian Association of Research Companies (where class A is the highest income)*.

### Quantitative Analysis Data

#### Non-response Data

The proportion of non-response had a systematic increase from question 60. Simple, clear questions with only two answer options (yes and no) had a non-response ratio between 1 and 6%. However, when the interviewee was confronted with a long list of options, such as very unlikely, unlikely, likely, or very likely, this ratio varied between 2 and 15%. The questions about height and weight had the highest percentage of non-responders in the entire questionnaire, 18 and 19%, respectively.

#### Agreement Data

Table 3 shows the internal agreement level by the Kappa index for primary outcomes. The agreement level was substantial (Kappa 0.60–0.79) or almost perfect (Kappa > 0.8) for almost all questions on the use of marijuana, alcohol, cigarettes, cocaine, crack, and binge drinking. Only the questions about the use in the last month had moderate reliability (Kappa between 0.55 and 0.60).

**Table 3:** Internal agreement level according to the Kappa index for the primary outcomes of drug use by public-school students from the city of São Paulo paired in the segments 1 and 2 of the #Tamojunto2.0 drug prevention program evaluation questionnaire (*N* = 207).

**Agreement for drug use**	**Kappa**	***p*-value**
**Use of alcohol**		
Life	0.8397	<0.001
Year	0.7607	<0.001
Month	0.5510	<0.001
**Binge drinking**		
Life	0.6067	<0.001
Year	0.6380	<0.001
Month	0.3383	<0.001
**Use of cigarettes**		
Life	0.7871	<0.001
Year	0.7171	<0.001
Month	0.5399	<0.001
**Use of inhalants**		
Life	0.5285	<0.001
Year	0.6463	<0.001
Month	0.4781	<0.001
**Use of marijuana**		
Life	0.8024	<0.001
Year	0.8791	<0.001
Month	0.6611	<0.001
**Use of cocaine**		
Life	0.7951	<0.001
Year	0.7449	<0.001
Month	0.5665	<0.001
**Use of crack**		
Life	0.6644	<0.001
Year	0.6644	<0.001
Month	0.4987	<0.001

The Construct Validity and Fit of the dimensional models of secondary outcomes are presented in Table 4. The model fit indices for the bullying, electronic games, psychosis screening, and damage due to alcohol use indicated that our model, constituted by observed and latent variables, has a good fit adjustment. However, in the initial model for the eating disorder symptoms, some of the fit indices didn't show adequade values. By inspecting the modification indices (MI), we found that the models fit would be improved with the association of the following observed measures: S5 with S1 (S5: Would you say that Food dominates your life?/S1: Do you make yourself Sick because your feel uncomfortably full?). These modifications improved the unidimensional model. The same occurred with the psychiatric symptoms' scale, but, in this case, even with the modification indices, the final models' fit was not good.

**Table 4:** The construct validity and fit of the dimensional models of secondary outcomes by public-school students from the city of São Paulo paired in the segments 1 and 2 of the #Tamojunto2.0 drug prevention program evaluation questionnaire.

	**RMSEA** ** estimate**	**RMSEA** ** probability**	**CFI**	**TLI**	**WRMR**
**Secondary outcomes**					
Bullying	0.025	0.996	0.952	0.945	1.193
Eating disorder symptoms	0.090	0.088	0.790	0.579	1.213
Eating disorder symptoms^*^	0.000	0.848	1.000	1.086	0.345
Psychiatric symptoms	0.052	0.357	0.695	0.666	2.276
Psychiatric symptoms^**^	0.050	0.485	0.716	0.688	2.203
Electronic games	0.030	0.820	0.994	0.992	0.951
Psychosis screening	0.000	0.690	1.000	1.011	0.108
Damage due to alcohol use	0.000	0.991	1.000	1.004	0.720

* *association of the observed measures S5 with S1 (S5: Would you say that Food dominates your life?/S1: Do you make yourself Sick because your feel uncomfortably full?)*.

** *association of the observed measures SDQ25 with SDQ7 (SDQ25: I manage to finish the activities I start. I can pay attention./SDQ7: I am generally obedient and usually do what adults ask me to)*.

Questions related to secondary outcomes had good reliability (Cronbach's alpha higher than 0.7) for all outcomes in both segments, except for the SCOFF, which had a weak consistency (Cronbach's alpha lower than 0.600) in both segments (Table 5).

**Table 5:** Cronbach's alpha for the secondary outcomes of drug use by public-school students from the city of São Paulo paired in the segments 1 and 2 of the #Tamojunto2.0 drug prevention program evaluation questionnaire.

**Secondary outcomes**			**Cronbach's alpha**	
	**Segment 1**	Segment 2^*^	
Bullying victimization	0.704	0.759	
Bullying practice	0.610	0.867	
SCOFF	0.402	0.517	
SDQ	0.755	0.777	
Excessive use of computer games	0.766	0.833	

*SCOFF, Sick/Control/One stone/Fat/Food; SDQ, Strengths and difficult questionnaire*.

* *15/20 days after baseline*.

### Qualitative Analysis Data

#### Direct Participatory Observation in the Classroom (Recorded on Field Sheets)

As for environment and logistics characteristics that facilitated or hindered questionnaire answering, potential barriers to questionnaire administration were identified in some classes. The students' misconduct and the use of cell phones in the classroom were notable factors related to the non-fulfillment of the task within the stipulated time (50 min). As for difficulties related to the structure and layout of the questions, the creation of secret codes in some classes lasted longer than expected (more than 5 min) due to understanding difficulties reported by some students during the task. In addition, semantic difficulties were also reported by students and registered in the field card by the administrators, such as ambiguous language, lack of concept or word understanding, and complex argumentation in some questions. For example, some questions about bullying raised questions because the students did not understand if they referred to the perpetrator or the victim. In addition, some students delivered the questionnaire with unanswered questions reporting “fatigue.”

There was a mean absence among enrolled students of 30%. According to the report of one of the teachers “a large number of absences is common,” especially on Fridays, the day where it can reach more than 50% among the students enrolled in the 8th year.

#### Focus Groups

Focus group data confirmed most of the problems recorded on field cards. As for the semantic barriers, it was observed that some words generated interpretation difficulties, such as the term “silhouette,” which was interpreted by the students as both “the whole body” and “just the part of the waist.” The name SABESP (Basic Sanitation Company of the State of São Paulo), which appeared as an example of water and sewage network in the ABEP socioeconomic module, also raised questions, and many students did not understand that it was related to “piped water.” In the question related to drug beliefs, a negative statement (“Smoking marijuana is not addictive”), also raised questions among many students since they had to choose “yes” or “no” if they agreed or disagreed with the statement, that is, a question with a negative response seemed to confuse the students. As for difficulties related to the layout and structure of the questions, the four-to-five points Likert-scale used in many issues was an important source of questions because the students could hardly differentiate between “unlikely” and “very unlikely,” suggesting a reduction to make the options more objective; “I thought it could have used another word, like right or wrong,” reported one of the students.

The time used to complete the questionnaire was 70 min, 20 min more than the time recommended by the directors, who authorized questionnaire application for one class period (50 min).

Finally, the more inconsistent questions were the ones in the psychosis module, since the students wrongly understood the statements about seeing and hearing what the others do not as being able to see and hear.

## Discussion

This study evaluated the quality of a questionnaire built from other instruments already in use nationally and internationally, which will be applied in the RCT to evaluate the effectiveness of the # Tamojunto2.0 program and could be applied in the evaluation of other school-based drug use prevention programs. The semantic validity, construct valididy and reliability of this questionnaire were investigated using qualitative and quantitative research methods with three data sources (structured questionnaire, focus groups, and field cards). It evidenced that, like in previous studies (Beaton et al., 2000), this data source triangulation had high consistency levels between results and greater information complementarity, allowing a more meaningful understanding and clarification of difficulties related to the development process of the instrument and facilitating the adequacy of the final version of the questionnaire to be applied.

The findings related to response reliability, assessed by the respondents' ability to give the same answers in different instrument administrations, proved to be positive in most questions. Questions related to the main outcome, drug use by adolescents, presented a Kappa index that showed a substantial or almost perfect agreement level, corroborating Komro et al. (2004). In addition, the questionnaire provides a better understanding of drug-related issues than the one analyzed by Prado et al. (2016), which aimed to evaluate the Unplugged Program and presented a moderate agreement level. Only the question that referred to drug use in the previous month had moderate reliability. However, this finding may have occurred due to the period described in the question (30 days) and the interval between one collection and another (between 15 and 20 days). Therefore, part of the retest period does not overlap with the test and may have generated different responses, although correct.

The Construct Validity and Fit of the dimensional models of secondary outcomes had a good fit adjustment. Only for psychiatric symptoms scale that the final models' fit was not good, but as it is a widely used scale with many validations, both in Brazil and internationally (Goodman, 1997; Goodman et al., 2003; Goodman and Goodman, 2009), we decided to keep it and analyze it better when we apply in a larger sample. Moreover, the results related to the reliability of the scales used to evaluate the secondary outcomes analyzed by the Cronbach's alpha showed good consistency, corroborating other studies assessing similar scales (Lemmens et al., 2015). This shows that the questionnaires included to evaluate these outcomes were well-understood by the students, thus, being reliable for this evaluation. Only the SCOFF questionnaire showed weak consistency in both segments. However, some authors argue that a Cronbach's alpha between 0.5 and 0.6 can be acceptable, provided that the results obtained with the instrument are cautiously treated (Maroco and Garcia-Marques, 2006). Therefore, it was decided to keep this scale in the final instrument because the model fit indices had a good fit and this questionnaire has already been validated in several countries (Kutz et al., 2020), in addition, it is a practical instrument and reduced size.

The analysis of the focus group audios confirmed the difficulties observed in the field cards and showed the need to replace some words. The word “silhouette” in the question about body image will need to be replaced by “figure” to make it clear what it refers to. Likert-scale questions, such as “very unlikely,” “unlikely,” “probable,” and “very likely,” need to be replaced by “no” and “yes” since high-quality questionnaires must prioritize simple sentences and avoid using difficult words or asking more than one question per sentence (Terwee et al., 2007). The low level of literacy in Brazil can, to some degree, be related to poor understanding of the questions, since the data from the International Student Assessment Program (PISA) showed that the performance of Brazilian students was below average in the reading competence test compared to students from other countries (INEP, 2015).

A high rate of non-responses, generally above 20%, generates results that are not representative of the population (Stavseth et al., 2019). Therefore, the final version of the questionnaire excluded questions about height and weight, as they had the highest percentage of non-responses of the entire questionnaire due to students' lack of information regarding these data. It is important to note that even with this exclusion, body dissatisfaction can still be assessed using the Stunkard Silhouette scale (Scagliusi et al., 2006). In addition, there was a systematic increase in non-responses from question 60, and due to criticisms about the length of the questionnaire and students' tiredness, who answered it without attention and care, the new questionnaire should be reduced by 20% of the questions.

The combination of field card and focus group evaluations with Kappa analyses, CFA and Cronbach's alpha test/retest was especially important to identify the necessary changes to this questionnaire. After these changes are made, the questionnaire will be suitable to be used in the #Tamojunto2.0 program effectiveness study.

## Data Availability Statement

The raw data supporting the conclusions of this article will be made available by the authors, without undue reservation.

## Ethics Statement

This study was approved by the Ethics Committee of the Federal University of São Paulo, number 2.806.301, and registered in the REBEC (Clinical Trials Registry of the Ministry of Health)—RBR-8cnkwq. Written informed consent from the participants' legal guardian/next of kin was not required to participate in this study in accordance with the national legislation and the institutional requirements.

## Author Contributions

PG was responsible for the first writing of this article and also, for the final version. JV and JNM contributed to the writing of the article and in the qualitative analyzes and HC-M with the quantitative and sample analyzes. SC, JJM, and MM revised the final version of the article. ZS in addition to revising the final version of the article and responsible for the study design. All authors have read and approved the manuscript.

## Conflict of Interest

The authors declare that the research was conducted in the absence of any commercial or financial relationships that could be construed as a potential conflict of interest.

## References

[B1] ABEP A. B. de empresas de pesquisa (2018). Economic classification criterion Brazil. Abep 1, 1–5. Available online at: http://www.abep.org/criterio-brasil

[B2] American Psychiatric Association (2013). Diagnostic and Statistical Manual of Mental Disorders, Fifth Edn. 10.1176/appi.books.9780890425596

[B3] Beaton D. E. Bombardier C. Guillemin F. Ferraz M. B. (2000). Guidelines for the process of cross-cultural adaptation of self-report measures. Spine 25, 3186–3101. 10.1097/00007632-200012150-00014 11124735

[B4] Carlini E. L. A. Noto A. R. Sanchez Z. Carlini C. M. A. Locatelli D. P. Abeid L. R.. (2010). VI Levantamento nacional sobre o consumo de drogas psicotrópicas entre estudantes do ensino fundamental e médio das redes pública e privada de ensino nas 27 capitais brasileiras. Brasilia/DF: SENAD - Secretaria Nacional de Políticas sobre Drogas.

[B5] Creswell J. (2009). Researchdesign: Qualitative, Quantitative and Mixed Methods Approaches. (3rd ed.). Los Angeles, CA; London; New Delhi; Singapore; Washington: Sage Publications.

[B6] Faggiano F. Galanti M. R. Bohrn K. Burkhart G. Vigna-Taglianti F. Cuomo L.. (2008). The effectiveness of a school-based substance abuse prevention program: EU-Dap cluster randomised controlled trial. Prev. Med. 47, 537–543. 10.1016/j.ypmed.2008.06.018 18657569

[B7] Faggiano F. Vigna-Taglianti F. Burkhart G. Bohrn K. Cuomo L. Gregori D.. (2010). The effectiveness of a school-based substance abuse prevention program: 18-Month follow-up of the EU-Dap cluster randomized controlled trial. Drug Alcohol Depend. 108, 56–64. 10.1016/j.drugalcdep.2009.11.018 20080363

[B8] Fidalgo T. M. Sanchez Z. M. Caetano S. C. Maia L. O. Carlini E. A. Martins S. S. (2016). The association of psychiatric symptomatology with patterns of alcohol, tobacco, and marijuana use among Brazilian high school students. Am. j. Addict. 25, 416–425. 10.1111/ajad.12407 27437619

[B9] Goodman A. Goodman R. (2009). Strengths and difficulties questionnaire as a dimensional measure of child mental health. J. Am. Acad. Child Adolesc. Psychiatry 48, 400–403. 10.1097/CHI.0b013e3181985068 19242383

[B10] Goodman R. (1997). The Strengths and difficulties questionnaire: a research note. J. Child Psychol. Psychiatry 38, 581–586. 9255702 10.1111/j.1469-7610.1997.tb01545.x

[B11] Goodman R. Meltzer H. Bailey V. (2003). The strengths and difficulties questionnaire: a pilot study on the validity of the self-report version. Int. Rev. Psychiatry 15, 173–177. 10.1080/0954026021000046137 12745329

[B12] IBGE (2016). Pesquisa Nacional de Saúde do Escolar 2015 (Instituto Brasileiro de Geografa e Estatística. Rio de Janeiro.

[B13] INEP (2015). Programme for International Students Assessments (PISA). Resumo de Resultados Nacionais Do PISA.

[B14] Komro K. A. Perry C. L. Munson K. A. Stigler M. H. Farbakhsh K. (2004). Reliability and validity of self-report measures to evaluate drug and violence prevention programs. J. Child Adolesc. Subst. Abuse 13, 17–51. 10.1300/J029v13n03_02

[B15] Kutz A. M. Marsh A. G. Gunderson C. G. Maguen S. Masheb R. M. (2020). Eating disorder screening: a systematic review and meta-analysis of diagnostic test characteristics of the SCOFF. J. Gene. Internal Med. 35, 885–893. 10.1007/s11606-019-05478-6 31705473 PMC7080881

[B16] Landis J. R. Koch G. G. (2012). The measurement of observer agreement for categorical data. Biometrics 33, 159–174. 10.2307/2529310 843571

[B17] Lemmens J. S. Patti M. Valkenburg Douglas A. Gentile. (2015). The internet gaming disorder scale. psychological assessment. Am. Psychol. Assoc. 15, 1040–3590. 10.1037/pas0000062 25558970

[B18] Little T. D. Rhemtulla M. Gibson K. Schoemann A. M. (2013). Why the items versus parcels controversy needn't be one. Psychol. Methods 18, 285–300. 10.1037/a0033266 23834418 PMC3909043

[B19] Maroco J. Garcia-Marques T. (2006). Portfolio evaluation of academic patent: a proposal to Brazil. Alpha Crombach Tabela Portugal 4, 65–90. 10.14417/lp.763

[B20] Morgan J. F. Reid F. Lacey J. H. (1999). The SCOFF questionnaire: assessment of a new screening tool for eating disorders. Bmj 319, 1467–1468. 10.1136/bmj.319.7223.1467 10582927 PMC28290

[B21] Neusa Maria Costa Alexandre and Marina Zambon Orpinelli Coluci (2011). Validade de conteúdo nos processos de construção e adaptação de instrumentos de medidas. Ciência e Saúde Coletiva 16, 3061–3068. 10.1590/S1413-81232011000800006 21808894

[B22] Newton N. C. Stapinski L. Slade T. Champion K. E. Barrett E. L. Chapman C.. (2018). Pathways to prevention: Protocol for the CAP (climate and preventure) study to evaluate the long-term effectiveness of school-based universal, selective and combined alcohol misuse prevention into early adulthood. BMC Public Health 18, 1–10. 10.1186/s12889-018-5554-y 29783974 PMC5963131

[B23] Prado M. C. D. O. Schneider D. R. Sañudo A. Pereira A. P. D. Horr J. F. Sanchez Z. M. (2016). Transcultural adaptation of questionnaire to evaluate drug use among students: The use of the EU-dap european questionnaire in Brazil. Substance Use Misuse 51, 449–458. 10.3109/10826084.2015.1117108 26894657

[B24] Sanchez Z. M. Valente J. Y. Sanudo A. Pereira A. P. D. Schneider D. R. Andreoni S. (2018). Effectiveness evaluation of the school-based drug prevention program #Tamojunto in Brazil: 21-month follow-up of a randomized controlled trial. Int. J. Drug Policy 60, 10–17. 10.1016/j.drugpo.2018.07.006 30081337

[B25] Scagliusi F. B. Alvarenga M. Polacow V. O. Cordás T. A. de Oliveira Queiroz G. K. Coelho D.. (2006). Concurrent and discriminant validity of the Stunkard's figure rating scale adapted into Portuguese. Appetite 47, 77–82. 10.1016/j.appet.2006.02.010 16750589

[B26] Solberg M. E. Olweus D. (2003). Prevalence estimation of school bullying with the olweus bully/victim questionnaire. Aggress. Behav. 29, 239–268. 10.1002/ab.10047

[B27] Stavseth M. R. Clausen T. Røislien J. (2019). How handling missing data may impact conclusions: A comparison of six different imputation methods for categorical questionnaire data. SAGE Open Med. 7:205031211882291. 10.1177/2050312118822912 30671242 PMC6329020

[B28] Stewart D. W. Shamdasani P. N. Rook D. W. (2007). Grupos focais, 2nd Edn. SAGE Publications, Ltd. 10.4135/9781412991841

[B29] Strauss A. Corbin J. (2008). Pesquisa Qualitativa: Técnicas e Procedimentos Para o Desenvolvimento de Teoria Fundamentada (2nd ed.). Porto Alegre: Artmed.

[B30] Strøm H. K. Adolfsen F. Fossum S. Kaiser S. Martinussen M. (2014). Effectiveness of school-based preventive interventions on adolescent alcohol use: a meta-analysis of randomized controlled trials. Subst. Abuse Treatment Prevent. Policy 9:48. 10.1186/1747-597X-9-48 25495012 PMC4274678

[B31] Temel G. Erdogan S. (2017). Determining the sample size in agreement studies. Marmara Med. J. 30, 101–112. 10.5472/marumj.344822

[B32] Terwee C. B. Bot S. D. M. de Boer M. R. van der Windt D. A. W. M. Knol D. L. Dekker J.. (2007). Quality criteria were proposed for measurement properties of health status questionnaires. J. Clin. Epidemiol. 60, 34–42. 10.1016/j.jclinepi.2006.03.012 17161752

[B33] Van Der Kreeft P. Wiborg G. Galanti M. R. Siliquini R. Bohrn K. Scatigna M.. (2009). ‘Unplugged': a new European school programme against substance abuse. Drugs 16, 167–181. 10.1080/09687630701731189

